# Acaricidal, Insecticidal, and Nematicidal Efficiency of Essential Oils Isolated from the *Satureja* Genus

**DOI:** 10.3390/ijerph18116050

**Published:** 2021-06-04

**Authors:** Asgar Ebadollahi, Jalal Jalali Sendi, Masumeh Ziaee, Patcharin Krutmuang

**Affiliations:** 1 Department of Plant Sciences, Moghan College of Agriculture and Natural Resources, University of Mohaghegh Ardabili, Ardabil 56199-36514, Iran; 2 Department of Plant Protection, Faculty of Agricultural Sciences, University of Guilan, Rasht 41635-1314, Iran; jjalali@guilan.ac.ir; 3 Department of Plant Protection, Faculty of Agriculture, Shahid Chamran University of Ahvaz, Ahvaz 61357-43311, Iran; m.ziaee@scu.ac.ir; 4 Department of Entomology and Plant Pathology, Faculty of Agriculture, Chiang Mai University, Chiang Mai 50200, Thailand; 5 Innovative Agriculture Research Center, Faculty of Agriculture, Chiang Mai University, Chiang Mai 50200, Thailand

**Keywords:** biopesticides, essential oil, multiple modes of action, *Satureja*, terpenes

## Abstract

The overuse of synthetic pesticides in plant protection strategies has resulted in numerous side effects, including environmental contamination, food staff residues, and a threat to non-target organisms. Several studies have been performed to assess the pesticidal effects of plant-derived essential oils and their components, as partially safe and effective agents, on economically important pests. The essential oils isolated from *Satureja* species are being used in medicinal, cosmetic, and food industries. Their great potential in pest management is promising, which is related to high amounts of terpenes presented in this genus. This review is focused on the acute and chronic acaricidal, insecticidal, and nematicidal effects of *Satureja* essential oil and their main components. The effects of eighteen *Satureja* species are documented, considering lethality, repellency, developmental inhibitory, and adverse effects on the feeding, life cycle, oviposition, and egg hatching. Further, the biochemical impairment, including impairments in esterases, acetylcholinesterase, and cytochrome P450 monooxygenases functions, are also considered. Finally, encapsulation and emulsification methods, based on controlled-release techniques, are suggested to overcome the low persistence and water solubility restrictions of these biopesticides. The present review offers *Satureja* essential oils and their major components as valuable alternatives to synthetic pesticides in the future of pest management.

## 1. Introduction

Although synthetic chemicals have been considered as the pest management strategy so far, their overuse has led to several side effects. These include soil and groundwater pollution, toxic residues on the food stuffs, pest resistance, outbreak of secondary pests, and harmful effects on non-target organisms such as fish, bees, predators, and parasites [1,2,3,4].

The plant essential oils as low-risk agents are recommended alternatives to chemical pesticides [5,6]. Essential oils are complex mixtures of aromatic and aliphatic compounds, which mainly consist of hydrocarbon monoterpenes, monoterpenoids, hydrocarbon sesquiterpenes, and sesquiterpenoids, and can be made by all plant parts, such as flowers, seeds, leaves, stems, and bark [7]. Essential oils are composed by plants as secondary metabolites with anti-herbivore activity, resulted in critical defense strategies against herbivorous pests along with other significant roles, such as allelopathic plant–plant interactions and attraction of pollinators [8]. Hence, the possibilities of pest resistance to plant-derived essential oils is very low [9]. Along with multiple modes of action and efficiency against a wide range of arthropod pests, essential oils also exhibit comparative lower toxicity on non-target organisms, such as mammals and beneficial insects compared to chemicals [10]. Additionally, with about 24–48 h half-lives, they are degraded quickly by natural degradation mechanisms and considered as biodegradable agents [9]. The pesticidal effects of essential oils isolated from several species of plant families, such as Lamiaceae, Asteraceae, Myrtaceae, Apiaceae, Cupressacae, and Rutaceae, against diverse groups of agricultural pests have been well-endorsed in recent years [11,12,13]. Along with the toxicity of plant essential oils to arthropod pests, there are promising findings against pathogenic nematodes [14,15].

The genus *Satureja* belongs to the Lamiaceae family, Nepetoidae subfamily, and the Mentheae tribe, that includes about 200 species of aromatic herbs and shrubs. They are broadly distributed in America, the Mediterranean area, Middle East, North Africa, and West Asia [16]. Several species from this genus, conventionally known as savory, especially summer savory (*Satureja hortensis* L.), are cultivated in various countries [17]. These aromatic plants possess a high content of essential oil (even about 4%) located in their leaves, stems, and flowers [18]. Numerous medicinal properties, including reduction of blood pressure, joint pains, rheumatic pains, stomachache, toothache, fever, diarrhea, dyspepsia, gastrointestinal bloating, influenza, colds, scabies and itching, eye strengthening, antioxidant, antidiabetic, and antimicrobial properties, of *Satureja* species, especially their extracted essential oils, are well-documented in the literature [16,19,20,21].

The present review aimed to update the current knowledge on the essential oils extracted from different *Satureja* species in controlling economically damaging insects, mites, ticks, and nematodes. Thus, vast amounts of individual research have been gathered from scientific databases, including Scopus, Web of Science, PubMed, and Google Scholar. Our main aim was to introduce a novel, safe, and efficient bio-rational agent(s), as alternatives to the detrimental chemicals. The search also considers the sub-lethal and biochemical changes after application of these compounds in order to obtain a thorough insight into their mode of action.

## 2. Pesticidal Effects of Essential Oils Extracted from Various *Satureja* Species

The great potential of several species from the *Satureja* genus, including *S. aintabensis* Davis, *S. bachtiarica* Bung, *S. cilicica* Davis, *S. cuneifolia* Ten, *S. hellenica* Halásky, *S. hortensis* L., *S. intermedia* C. A. Mey, *S. isophylla* L., *S. khuzestanica* Jamzad, *S. montana* L., *S. parnassica* Heldr & Sart ex Boiss, *S. parvifolia* (Phil) Epling, *S. rechingeri* Jamzad, *S. sahendica* Bornm, *S. spicigera* Boiss, *S. spinosa* L., *S. thymbra* L., and *S. wiedemanniana* (Avé-Lall) Velen, has been reported in the insects, mites, ticks, and nematodes’ management. As shown in Table 1, the efficiency of *Satureja* essential oils was assessed against a diverse group of insects from Coleoptera to Diptera, Hemiptera, Homoptera, Lepidoptera, Phthiraptera, and Thysanoptera orders, and similarly, on other arthropods, including mites and ticks, and plant pathogenic nematodes.

The pesticidal effects of *Satureja* essential oils can be considered from two viewpoints, i.e., lethal and sub-lethal. For example, along with acute fumigant toxicity of *S. thymbra* essential oil against the adults of *Acanthoscelides obtectus*, *Ephestia. kuehniella*, and *Leptinotarsa decemlineata*, its repellent effect on *Aedes albopictus* was also reported [22,23,24]. In general, there are several sub-lethal bio-efficiencies of *Satureja* essential oils, including repellent and antifeedant activities and adverse effects on fecundity, fertility, and life cycle. Some of these studies have also considered the biochemical mode of action in pests such as general esterase, acetylcholinesterase, and cytochrome P450 monooxygenases [25,26,27]. The studies include different developmental stages of pests, from eggs to larvae, pupae, and adults. Among the large species of *Satureja* studied, the essential oils of *S. hortensis*, *S. montana*, and *S. thymbra* are considered as the most promising in pest management (Table 1). Another prospective is the possibility of using *Satureja* essential oil along with other pest control agents, such as entomopathogenic fungi. For example, Hosseinzadeh et al. [28] indicated that the essential oil of *S. sahendica* had a significant synergistic effect with entomopathogenic fungus *Beauveria bassiana* against the cowpea weevil, *Callosobruchus maculatus* (Fabricius).

**Table 1:** Reported acaricidal, insecticidal, and nematicidal effects of the essential oils isolated from different *Satureja* species.

Pests	*Satureja* Species	Bioassay and Target Pest	Efficiency
Insects	*S. aintabensis* Davis	Contact assay (on treated filter papers) against the adult females of the turnip aphid (*Lipaphis pseudobrassicae* (Davis)).	Significant toxicity with LC_50_ (lethal concentration to kill 50% of tested insects) of 1.7 mg/mL after 1 h [29].
	*S. bachtiarica* Bung	Aqueous suspension of essential oil against the third- and fourth-instar larvae of the Asian malaria mosquito (*Anopheles stephensi*) and filariasis vector (*Culex quinquefasciatus* Say).	The larval mortality of 100% at the concentration of 160 ppm after 24 h [30].
		Fumigant and repellency assays (by impregnated filter papers in glass vials and Petri dishes, respectively) against the adults of red flour beetle (*Tribolium castaneum* (Herbst)).	Significant fumigant toxicity (LC_50_ = 4.71 mg/L) and repellent action (100% at the concentration of 1% *v*/*v* after 8 h) [31].
		Fumigant assay (by impregnated filter papers) against the fourth-instar larvae of tomato leafminer (*Tuta absoluta* (Meyrick))	Significant fumigant toxicity (LC_50_ = 25.03 µL/L) and reduction in activity of general esterases (α and β) (*p* < 0.05) [25].
	*S. cilicica* Davis	Contact assay (on treated filter papers) against the Colorado potato beetle (*Leptinotarsa decemlineata* Say).	High mortality of the first (97.7%), second (95.5%), third (91.1%), and fourth (97.7%) instar larvae and the adults (84.4%) at 20 µL/cm^2^ after 96 h [24].
	*S. cuneifolia* Ten	Fumigant assay (by impregnated filter papers) on field-collected sand flies (Diptera: Psychodidae: Phlebotomie).	The knockdown rate of 100% at the concentration of 20.0 µL/L after 0.5 h [32].
		Contact assay (on treated filter papers) against *L. decemlineata*.	High mortality of the first (93.3%), second (91.1%), third (95.5%), and fourth (88.8%) instar larvae and the adults (86.6%) at 20 µL/cm^2^ after 96 h [24].
	*S. hortensis* L.	Aqueous suspension of essential oil against the larvae of the *C. quinquefasciatus*.	Significant toxicity (LC_50_ = 36.0 μg/mL), the reduction in the adult emergence by a quarter of the control (*p* < 0.05), and 100% oviposition deterrence by the concentration of 200 ppm [33].
		Fumigant assay (by impregnated filter papers) against the adults of bean weevils (*Bruchus dentipes* (Baudi)).	The mortality of 100% at the concentration of 20.0 µL/L after 24 h [34]
		Fumigant assay (by impregnated filter papers) against the cotton whitefly (*Bemisia tabaci*) on the eggplant leaves.	The 100% mortality of adult females at 2.4 mL/cm^3^ of essential oil after 24 h [35].
		Fumigant assay (by impregnated filter papers) against the adults of *B. tabaci* on cucumber leaves.	The mortality of 100% at 2 µL/L of essential oil after 12 h [36].
		Contact assay (on treated filter papers) against the adults of *C. maculatus*.	Toxic to the adults with LC_50_ values of 5.36 and 6.41 µL/cm^2^ on the males and females, respectively [37].
		Fumigant assay (by impregnated filter papers) against the adults of *C. maculatus*.	The 91.2% adult mortality at 60 mL/L and the 94.5% egg mortality at 4.3 mL/L of essential oil after 24 h [38].
		Fumigant assay (by impregnated filter papers) against the adults of maize weevil (*Sitophilus zeamais* Motschulsky).	The 100% mortality at the concertation of 10 µL/L after 96 h exposure time [39].
		Leaf dipping method against the larvae of mulberry pyralid (*Glyphodes pyloalis* Walker)	Significant feeding inhibition (44.35% at the concentration of 0.025%), decrease in the amount of protein, lipid, carbohydrates, and the activity of α-amylase, esterase, and glutathione S-transferase (*p* < 0.05) [40].
		Antifeedant assay (by treated flour disk) on first-instar larvae of the Indian meal moth (*Plodia interpunctella* Hübner).	Significant reduction in the relative growth (0.01 mg/day) and consumption (0.31 mg/day) rates of larvae treated by 0.22 µL/cm^2^ of essential oil compared to control (0.05 and 0.10 mg/day, respectively) (*p* < 0.05) [41].
		In-vivo repellent assay (by counting the number of bites on the back of rabbits) against the adult females of *A. stephensi*.	A protection time of 4.16 h at ED_50_ (effective dose) of 5.63 mg/cm^2^ [42].
		Contact assay (by direct spraying) on the larvae of the American White Butterfly (*Hypantria cunea* Drury).	The 68.8% mortality of third- and fourth-instars larvae at 1.67 µL/cm^2^ after 96 h [43]
		Spraying on black chokeberry inflorescences ingested by the larvae of grey Knot-horn (*Acrobasis advenella* (Zinck)).	Significant reduction in the amount of α- and β-glucosidase of treated larvae and the emergence and longevity of adults [17].
		Fumigant assay (by impregnated filter papers) on the third-instar larvae of Mediterranean flour moth (*Ephestia kuehniella* Zeller).	A mortality of 88.3% at 60 µL/L after 24 h (LC_50_ = 30.09 µL/L) [44].
		Oviposition deterrence and feeding-site assays (by choice test with treated black chokeberry infructescences) on *A. advenella*.	Significant reduction in laid eggs (3.89%) and feeding site of larvae (27.35%) compared to control groups (17.15% and 4.69%, respectively) [45].
		Fumigant assay (by impregnated filter papers) against the adults of lesser grain borer (*Rhyzopertha dominica* (Fabricius)) and *T. castaneum*.	Significant toxicity against both insects with LC_50_ values of 16.47 and 25.75 µL/L after 72 h, respectively [46].
	*S. intermedia* C. A. Mey	Fumigant assay (by impregnated filter papers) against the adults of saw-toothed beetle (*Oryzaephilus surinamensis* (L.)), *R. dominica*, the khapra beetle (*Trogoderma granarium* Everts), and *T. castaneum*, and contact assay (leaf dipping method) on the adult female of the oleander aphid (*Aphis nerii*).	High fumigant and contact toxicity against all pests with LC_50_ values of 8.15, 12.83, 2.49, and 35.61 µL/L, and 418.38 µg/mL, respectively [47].
	*S. isophylla* L.	Fumigant assay (by impregnated filter papers) against cabbage aphid (*Brevicoryne brassica* L.) and black bean aphid (*Aphis fabae* Scop) on acacia leaves.	Significant fumigant toxicity against both insects with LC_50_ values of 7.33 and 14.29 µL/L, respectively [48].
		Fumigant assay (by impregnated filter papers) against *A. fabae* on acacia leaf.	Significant fumigant toxicity against adult females (LC_50_ = 14.29 µL/L) and nymph production detergency at 8.53 µL/L (*p* < 0.05) [49].
		Fumigant assay (by impregnated filter papers) against the adults of *R. dominica* and *T. castaneum*.	High mortality of *R. dominica* (98.7%) and *T. castaneum* (90.0%) at 35.3 and 55.0 µL/L concentrations respectively, after 72 h [50].
	*S. khuzestanica* Jamzad	In vivo mosquito repellents assay for human skin (from elbow to wrist) against the adults of *A. stephensi*.	Significant reduction in the number of mosquito bites compared to the control group (*p* < 0.01) [51].
		Toxicity assay (by impregnated potato leaves in Petri dishes) on the adults of *L. decemlineata*.	Significant mortality of the fourth-instar larvae and adults with LC_50_ values of 23.36 and 167.96 ppm, respectively [52].
		Fumigant and repellent assays (by impregnated filter papers in glass vials and Petri dishes, respectively) against the adults of *T. castaneum*.	Significant fumigant toxicity (LC_50_ = 2.51 mg/L) and repellent action (100% at the concentration of 1% *v*/*v* after 8 h) [31].
		Fumigant assay (by impregnated filter papers) against the fourth-instar larvae of *T. absoluta*.	Significant fumigant toxicity (LC_50_ = 17.51 µL/L) and reduction in activity of general esterases (α and β) (*p* < 0.05) [25].
	*S. montana* L.	Aqueous suspension of essential oil on the fourth-instar larvae of common house mosquito (*Culex pipiens* L.).	Significant larvicidal activity with LC_50_ value of 37.70 mg/L [53].
		Repellent assay (by treated green bean leaves in Petri dishes) on the Western flower thrips (*Frankliniella occidentalis*).	A complete repellency (100%) at the concentration of 2.0% after 1 h [54].
		Contact assay (topical application) against the fruit fly (*Drosophila suzukii* (Matsumura)).	Significant toxicity with LC_50_ values of 2.95 and 4.59 µg/fly on the male and female adults, respectively [26].
		Aqueous suspension of essential oil against the third-instar larvae of *C. quinquefasciatus*	High larvicidal effectiveness with LC_50_ value of 25.6 μL/L [55].
		Contact assay (on treated filter papers) against *L. decemlineata*.	High mortality of the first (100%), second (97.7%), third (95.5%), and fourth (97.7%) instar larvae and the adults (88.8%) at the concentration of 20 µL/cm^2^ after 96 h [24].
	*S. parnassica* Heldr & Sart ex Boiss	Aqueous suspension of essential oil on the fourth-instar larvae *C. pipiens*.	Significant larvicidal activity with LC_50_ value of 37.70 mg/L [53].
	*S. parvifolia* (Phil.) Epling	Fumigant assay (by impregnated filter papers) on the adult-females of the head louse (*Pediculus humanus capitis* De Geer).	Significantly toxic with KT_50_ value (time to 50% knockdown) of 36.06 min at 60 µL of essential oil concentration [56].
		Repellent assay (by treated filter papers in Petri dishes) against the nymphs of kissing bug (*Triatoma infestans* Klug).	The repellency of 100% and 76.0% at the concentration of 0.5% (*w*/*v*) after 1 and 24 h [57].
	*S. rechingeri* Jamzad	Fumigant and repellency assays (by impregnated filter papers in glass vials and Petri dishes, respectively) against the adults of *T. castaneum*.	Significant fumigant toxicity (LC_50_ = 3.27 mg/L) and repellent action (100% at the concentration of 1% *v*/*v*) after 8 h [31].
		Fumigant assay (by impregnated filter papers) against the fourth-instar larvae of *T. absoluta*.	Significant fumigant toxicity (LC_50_ = 34.33 µL/L) and reduction in activity of general esterases (α and β) (*p* < 0.05) [25].
	*S. sahendica* Bornm	Fumigant assay (by impregnated filter papers) against the adults of *C. maculatus*.	Significant toxicity with LC_50_ value of 22.42 µL/L [28].
	*S. spicigera* Boiss	Fumigant assay (by impregnated filter papers) against the adults of granary weevil (*Sitophilus granarius* (L.)).	The 94.27% mortality at the concentration of 20.0 µL/L after 86 h [58].
		Fumigant assay (by impregnated filter papers) against *S. zeamais*.	The mortality of 100% at concertation of 10 µL/L after 96 h exposure time [39].
		Contact assay (on treated filter papers) against *L. decemlineata*.	High mortality of the first (100%), second (100%), third (95.5%), and fourth (95.5%) instar larvae and the adults (80.0%) at 20 µL/cm^2^ after 96 h [24].
	*S. spinosa* L.	Aqueous suspension of essential oil on the fourth-instar larvae *C. pipiens*.	Significant larvicidal toxicity with LC_50_ value of 37.70 mg/L [53].
	*S. thymbra* L.	Aqueous suspension of essential oil on the fourth-instar larvae *C. pipiens*.	Significant larvicidal toxicity with LC_50_ value of 37.70 mg/L [53].
		Fumigant assay (by impregnated filter papers) against *E. kuehniella* and *P. interpunctella*.	The 100% egg mortality of *E. kuehniella* and *P. interpunctella* at 200 μL/L after 96 h [59].
		Fumigant assay (by impregnated filter papers) against the adults of *E. kuehniella*, *P. interpunctella*, and bean weevil (*Acanthoscelides obtectus* Say).	The 100% mortality of *E. kuehniella*, *P. interpunctella* (at 9 and 25 µL/L respectively, after 24 h), and *A. obtectus* (195 µL/L after 144 h) [22].
		Fumigant assay (by impregnated filter papers) against *E. kuehniella*.	Significant adulticidal toxicity (LC_50_ = 13.92 µL/L after 12 h) and reduction in the larval and adult emergence and egg production compared to control groups (*p* < 0.05) [60].
		Fumigant (by impregnated filter papers on the adults) and aqueous suspension (on the larvae) assays on African malaria mosquito (*Anopheles gambiae* Giles).	The 100% mortality of adults and larvae at 32.2 µg/mL and 3 mg/mL of essential oil respectively, after 24 h [61].
		Spraying on grape leaves against the nymphs and female adults of the vine mealybug (*Planococcus ficus* (Signoret)).	Significant mortality on nymphs (LC_50_ = 2.7 mg/mL) and adults (LC_50_ = 6.3 mg/mL) after 24 h [62].
		In vivo larvicidal assay in basins against the larvae of dengue vector (*Aedes albopictus* Skuse).	Significant larval mortality (96.00% at 29 mg/L of the essential oil) after 24 h [23].
		Contact assay (on treated filter papers) against *L. decemlineata*.	High mortality of the first (100.0%), second (95.5%), third (97.7%), and fourth (95.5%) instar larvae and the adults (97.7%) at 20 µL/cm^2^ after 96 h [24].
	*S. wiedemanniana* (Avé-Lall) Velen	Contact toxicity (on treated filter papers) against the adult females *L. pseudobrassicae*.	Significant toxicity with LC_50_ of 1.0 mg/mL after 1 h [29].
Mites and Ticks	*S. bachtiarica*	Fumigant (by impregnated filter papers) and repellency assays (by treated leaf discs) against the two-spotted spider mite (*Tetranychus urticae* Koch) in Petri dishes.	Significant fumigant toxicity (LC_50_ = 44.06 µL/L) and high repellent action at 44.06 µL/L after 24 h [27].
	*S. hortensis*	Fumigant assay (by impregnated filter papers) against *T. urticae* on fresh leaves of bean.	The 96.6% mortality of nymphs and adults of *T. urticae* at concentration of 3.13 µL/L after 96 h [63].
		Fumigant (by impregnated filter papers) and contact (leaf dipping method) assays on the adults of *T. urticae*.	Significant fumigant and contact toxicity with LC_50_ values of 7.074 μL/L and 0.876% (*v*/*v*), respectively [64].
		Fumigant assays (by impregnated filter papers) against *T. urticae* on bean leaves.	Significant toxicity against the adults and eggs with 24 h LC_50_ values of 1.44 and 1.31 µL/L [65].
	*S. khuzestanica*	Fumigant (by impregnated filter papers) and repellency assays (by treated leaf discs) against *T. urticae* in Petri dishes.	Significant fumigant toxicity (LC_50_ = 31.11 µL/L) and high repellent action at 18.85 µL/L after 24 h [27].
	*S. sahendica*	Fumigant assay (by impregnated filter papers) against *T. urticae* on bean leaf discs.	Significant adulticidal (24 h LC_50_ = 0.98 µL/L) and ovicidal (72 h LC_50_ = 0.54 µL/L) toxicity [66].
	*S. thymbra*	Fumigant assay (by treated cotton wick) on the adults of the Mediterranean tick (*Hyalomma marginatum*).	The complete mortality (100%) at 40.0 µL/L within 3 h [67].
Nematodes	*S. hellenica* Halácsy	Immersion of the cotton root-knot nematode (*Meloidogyne incognita* (Kofold & White)) and the root-knot nematode (*Meloidogyne javanica* (Treub)) in aqueous suspension of essential oil.	The 100% paralysis of the second-stage juveniles (J2) of both species at the concentration of 2000 µL/L after 96 h [68].
	*S. montana*	Immersion of the mixed stages of pine wood nematode (*Bursaphelenchus xylophilus* Nickle) in aqueous suspension of essential oil.	The 100% mortality of nematodes exposed to a 2 mg/mL solution after 24 h [69].
		Spraying of the aqueous suspension of essential oil on *B. xylophilus* co-cultured with *Pinus pinaster* shoot.	Significant decrease in the population growth of nematode compared to the control groups (*p* < 0.05) [70].
		Spraying of the aqueous suspension of essential oil on the Columbia root-knot nematode *(Meloidogyne chitwoodi* Golden) co-cultured with *Solanum tuberosum* hairy roots.	Significant decrease in the population growth of nematode compared to the control groups (*p* < 0.05) [71].

Furthermore, as shown in Table 1, in addition to agricultural pests, the acute toxicity and repellent action of *Satureja* essential oils against larvae and adults of blood-sucking mosquitos that carry pathogenic agents were also approved. For example, high susceptibility of the Asian malaria mosquito (*A. stephensi*) and the filariasis vector mosquito (*C. quinquefasciatus*) to the essential oil of *S. bachtiarica* was reported, in which 100% larval mortality of both insects was attained by the concentration of 160 ppm after 24 h exposure time [30].

## 3. Relationship between Compositions of *Satureja* Essential Oils with Pesticidal Properties

The major compounds of essential oils of different *Satureja* species’ insecticidal, acaricidal, and nematicidal activities are depicted in Table 2. Some compounds such as γ-terpinene, borneol, carvacrol, *p*-cymene, and thymol were identified in many species. For example, thymol with high percentage is the main compound of *S. aintabensis*, *S. bachtiarica*, *S. cilicica*, *S. intermedia*, *S. isophylla*, *S. montana*, *S. parnassica*, *S. sahendica*, *S. spinosa*, *S. thymbra*, and *S. wiedemanniana* essential oils. However, some compounds, such as estragole, piperitenone, piperitenone oxide, α-terpineol, β-caryophyllene, and β-myrcene, were recognized in a species: estragole in the *S. hortensis*, Piperitenone and piperitenone oxide in *S. parvifolia* essential oil, and β-myrcene in *S. isophylla* essential oil (Table 2).

The identified compounds in the essential oils of *Satureja* species are categorized in the monoterpene hydrocarbon, monoterpenoid, sesquiterpene hydrocarbon, sesquiterpenoid, and phenylpropanoid groups (see Table 3). Indeed, the majority of recognized compounds are in the monoterpene group, with lower molecular weight than others, and only three compounds belong to other categories. There is sufficient evidence that the monoterpenes, especially monoterpenoids, have high pesticidal properties, and some novel and reliable outcomes in this field are shown in Table 3. For example, the toxicity of thymol, as one of main components in several species of the *Satureja* genus, was reported against the African cotton leafworm (*Spodoptera littoralis* Boisduval), the bed bugs (*Cimex lectularius* L.), the Colorado potato beetle (*Leptinotarsa decemlineata* Say), the granary weevil (*Sitophilus granarius* (L.)), the green peach aphid (*Myzus persicae* (Sulzer)), and the root-knot nematode (*Meloidogyne javanica* (Treub) Chitwood) [73,76,77]. It can be concluded from these studies that the presence of higher total monoterpenoid content of essential oils had a positive correlation with their pesticidal activity [78,79,80,81]. Thus, the acaricidal, insecticidal, and nematicidal effects of *Satureja* essential oils may be related to the high amounts of compounds listed in Table 3. It was also demonstrated that the phenolic monoterpenoids such as thymol with CH(CH_3_)_2_ functional group displayed significantly higher pesticidal effects compared to other terpenes, such as carvacrol and eugenol with CH_3_ and OCH_3_ functional groups, respectively [82,83]. However, the synergistic acaricidal, insecticidal, and nematicidal effects of minor components such as α- and β-pinene, camphor, menthol, sabinene, and thujene should also be considered [84,85,86,87]. For instance, the synergistic insecticidal action of terpenes that have methyl functional groups such as *p*-cymene and limonene with borneol is another consideration already reported by Pavela [83].

## 4. Modes of Action of Essential Oils and Their Components

The acetylcholinesterase (AChE) is actively involved in metabolic conversion of ‘acetylcholine’ in the synaptic cleft of arthropods and has two catalytic and peripheral target sites. The insect-specific cysteine residue positioned at the acetylcholinesterase active site is a proposed target site for developing insecticides to reduce off-target toxicity [94]. On the other hand, inhibition of pest-specific acetylcholinesterase will decrease the risk of utilized pesticides on non-target organisms, such as mammals [94]. Some essential oils and compounds are reported to bind with these target sites to inhibit the AChE action [95,96,97]. Park et al. [26] revealed that the essential oil of *S. montana* had significant AChE inhibitory activity against the fruit fly (*Drosophila suzukii* (Matsumura)), along with high toxicity. The inhibition of AChE leads to acetylcholine accumulation, hyperactivity, paralysis, and death of the pest. Along with terpenes, the well-known phenylpropane estragole has also shown AChE inhibitory effects [98,99]. It should be noted that the AChE inhibition can occur in both contact and fumigation methods of used essential oils [100,101]. Octopamine, as a neurotransmitter, neuromodulator, and hormone, is one of the important biogenic amines in invertebrates and is released at times of high energy demands [102]. Octopamine receptor alteration is considered as another mode of action of essential oils or their components [103]. The blockage of gamma-amino butyric acid (GABA) and nicotinic acetylcholine (nAChR) receptors has also been documented in some studies [97,104].

Beside the neurotoxic modes of pesticidal action of essential oils and compounds, there are several studies indicating enzymatic and non-enzymatic effects. The destructive effects of essential oils and their compounds on esterases and glutathione S-transferases (GSTs) as imperative detoxifying enzymes in arthropod pests are reported [88,105,106]. Disruption of the function of detoxifying enzymes may reduce the probability of pest resistance [107], and this has been clearly depicted by essential oils and their components. Farahani et al. [27] showed that the essential oil of *S. khuzestanica* had adverse effects on cytochrome P450 monooxygenases (P450, responsible for the oxidative metabolism of a variety of xenobiotics and endogenous compounds) function of two spotted spider mites (*Tetranychus urticae* Koch), along with toxic and repellent activities. The adverse effects of these agents on digestive enzymes such as lipases, proteases, α-amylases, α-glucosidases, and β-glucosidases were also reported [106], which can be very effective in reducing the nutritional efficiency of pests. Effects on energy reservoirs of the pest by decreasing the protein, glucose, and triglyceride contents and disrupting the action of immunological and hematological parameters are the other reasons to approve the multiple modes of action of these eco-friendly bio-pesticides [108,109].

## 5. Proposed New Formulations for Greenhouse and Field Applications

Although great potential for acaricidal, insecticidal, and nematicidal activity of *Satureja* essential oils and compounds have been reported, limitations such as susceptibility to light, moisture, oxygen, and temperature may restrict their application in the pest management strategies [5]. Indeed, the use of essential oils and their components in non-crop agriculture in the management of stored product pests, flies, and cockroaches is effective [110]. Additionally, the larvicidal activity of essential oils by treating standing water and waterways and their repellent effects on adults may be useful in mosquito management (See Table 1 and Table 3 for examples). Due to the disadvantage of low persistence in environmental conditions, the application of essential oils in crop agriculture can be limited [6]. Soft body and sucking pests (viz., aphids, thrips, and mites) are usually controlled by essential oils on crops, particularly under low pest pressure [110]. For example, Western flower thrip and green peach aphid were successfully controlled by the essential oil-based insecticide Ectrol (EcotecTM, California, USA) on lettuce and strawberry. However, partial efficiency was achieved against larger chewing insect pests, such as coleopterans and lepidopterans [110].

Nanoencapsulation based on the controlled release technique has been offered to overcome the lack of persistence restriction of bio-pesticides [111]. In the nanoencapsulation process, the active agent as a solid, liquid, or gas is surrounded by a thin layer of natural or synthesized polymer or a membrane to keep the core active agent from harmful environmental factors [112]. Generally, reducing the amount of active ingredients and minimizing evaporation and its controlled release are main advantages of nanoencapsulation [111]. However, along with above-mentioned advantages, expensive and difficult processes of the creation of nano-formulations should be considered. In the study of Ahmadi et al. [65], encapsulation of *S. hortensis* essential oil in chitosan-tripolyphosphate nanoparticles improved its ovicidal and adulticidal toxicity against *T. urticae*. Along with high toxicity, nanoencapsulation of *S. hortensis* essential oil in chitosan-tripolyphosphate nanoparticles enhanced its persistence so that 80% and 15% mortality was achieved for nano-encapsulated and pure essential oil formulation after 14 days. Usha Rani et al. [113] evaluated the antifeedant activity of pure and silica nanoparticles-based capsulated α-pinene and linalool against the tobacco cutworm (*Spodoptera litura* F.) and the castor semi-looper (*Achaea janata* L.). Although both terpenes had significant antifeedant effects, nano-capsule formulation augmented their effectiveness up to 10 and 25 times for *A. Janata* and *S. litura*, respectively. The same results regarding the enhancing toxicity and persistence of other essential oils by encapsulation in polymeric and non-polymeric materials, such as poly(ethylene glycol), myristic acid-chitosan, and mesoporous material, were also documented [114,115,116]. The preparation of nano-emulsions is another applicable method to solve the solubility restriction of essential oils in water and is more effective with minute quantities of toxic substances, both in medicinal and agricultural pest management prospects [117,118]. Further, the combination of essential oils with other protectants such as microbial agents may enhance their effectiveness. For example, the combination of *S. sahendica* essential oil with entomopathogenic fungus *Beauveria bassiana* augmented its toxicity against cowpea weevil, and insect pest mortality increased from 50% after a 1-day exposure time to 80% after 7 days [28].

## 6. Conclusions

Along with antibacterial, antifungal, antiviral, and general importance in medicinal, food, and cosmetic industries [119,120,121], the essential oils isolated from different species of *Satureja* genus could have great potential in the management of detrimental mite and tick Acari, insects, and nematodes. Pesticidal effects of *Satureja* species essential oils, which may be commonly related to their main terpenes [67,83,86], were reported as lethal contact and fumigant toxicity to sublethal repellent action, developmental inhibitory effects, adverse effects on the feeding, life cycle, oviposition, and egg hatching, and biochemical disturbances, such as reduction in general esterase content and inhibition of acetylcholinesterase and cytochrome P450 monooxygenases functions (see Table 1 and Table 3). Such multiple modes of action of essential oils and their compounds, in addition to reducing pest resistance, can affect a wide range of pests [5,9]. Despite all of the mentioned advantages, high volatility or lack of persistence and insolubility in water are the main restrictions in the commercialization and extensive application of these compounds [110]. Accordingly, their application is principally focused against indoor non-crop pests such as storage pests, flies, and cockroaches [96,114]. Further, the acute toxicity against larvae and repellent activity on the adults of mosquitos that carry pathogens and suck blood were also documented in Table 1 and Table 3. However, with micro- and nano-encapsulation on the basis of controlled release techniques, their persistence can be increased [122]. Although nano-emulsification is also a suitable way to dissolve essential oils in water [123,124], it is possible to increase their effectiveness by combined application with microbial control agents, such as entomopathogenic fungi [28]. These less-toxic substances may help in agriculture and environmental protection and can be proposed to countries that apply extreme amounts of synthetic pesticides. However, effects on beneficial and non-target organisms, residues on food products, and more importantly, considering a method for lower cost of *Satureja* essential oils and their components, should also be investigated in future research.

## Figures and Tables

**Table 2:** Main components of the *Satureja* species essential oils documented as promising insecticidal, acaricidal, and nematicidal agents.

Essential Oil	Main Components
*S. aintabensis*	*p*-Cymene (33%) and thymol (32%) [29].
*S. bachtiarica*	Thymol (28.0%), caryophyllene oxide (17.0%), carvacrol (13.2%), borneol (11.6%), and linalool (9.6%) [31].
*S. cilicica*	Thymol (68.9%), *p*-cymene (7.8%), borneol (2.9%), and linalool (1.8%) [29].
*S. cuneifolia*	Carvacrol (48.7%), *p*-cymene (38.1%), α-terpineol (1.9%), and borneol (1.9%) [72].
*S. hellenica*	*p*-Cymene (27.46%), carvacrol (23.25%), and borneol (6.79%) [68].
*S. hortensis*	Estragole (82.1%), β-ocimene (11.9%), and limonene (2.3%) [46].
*S. intermedia*	Thymol (48.1%), carvacrol (11.8%), *p*-cymene (8.1%), and γ-terpinene (8.1%) [47].
*S. isophylla*	Thymol (41.5%), *p*-cymene (25.9%), γ-terpinene (16.9%), β-myrcene (2.1%), and α-terpinene (1.6%) [50].
*S. khuzestanica*	Carvacrol (48.0%), *p*-cymene (18.5%), and γ-terpinene (11%) [21].
*S. montana*	Carvacrol (58.3%), *p*-cymene (18.3%), γ-terpinene (9.2%), and thymol (4.8%) [73].
*S. parnassica*	Carvacrol (6.4%), thymol (44.4%), γ-terpinene (12.3%), *p*-cymene (8.4%), and β-caryophyllene (4.4%) [53].
*S. parvifolia*	Piperitenone oxide (67.3%), piperitenone (7.2%), and pulegone (1.9%) [74].
*S. rechingeri*	Carvacrol (82.5%), γ-terpinene (2.7%), *p*-cymene (2.6%), and terpinene-4-ol (2.0%) [31].
*S. sahendica*	*p*-Cymene (30.2%), thymol (29.6%), and γ-terpinene (27.7%) [75].
*S. spicigera*	Carvacrol (90.1%), *p*-cymene (4.1%), and γ-terpinene (2.6%) [29].
*S. spinosa*	Carvacrol (47.1%), thymol (12.4%), γ-terpinene (6.5%), *p*-cymene (5.5%), and β-caryophyllene (5.0%) [53].
*S. thymbra*	Carvacrol (57.1%), *p*-cymene (21.9%), thymol (8.0%), and γ-terpinene (4.4%) [29].
*S. wiedemanniana*	Carvacrol (40%) and thymol (14%) [29].

**Table 3:** Characteristics and pesticidal activities of main components identified in *Satureja* species.

Classification	Components	Structure	Formula	Molecular Weight (g/mol)	Pesticidal Activities
Monoterpene hydrocarbon	*p*-Cymene	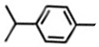	C_10_H_14_	134.22	The inhibition of acetylcholine esterase and insecticidal activity on the rice weevil (*Sitophilus oryzae* (L.)) [87].
	γ-Terpinene	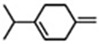	C_10_H_16_	136.23	Fumigant toxicity against the adults of the housefly (*Musca domestica* L.) [88].
	Limonene	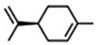	C_10_H_16_	136.23	Fumigant toxicity against the adults of *M. domestica* [88].
	α-Terpinene	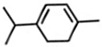	C_10_H_16_	136.23	The inhibition of acetylcholine esterase and insecticidal activity on *S. oryzae* [87].
	β-Myrcene	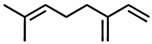	C_10_H_16_	136.23	The inhibition of acetylcholine esterase and insecticidal activity on *S. oryzae* [87].
	β-Ocimene	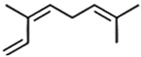	C_10_H_16_	136.23	Fumigant and contact toxicity, and acetylcholine esterase inhibition activity against the German cockroach (*Blattella germanica* (L)) [89].
Monoterpenoid	Carvacrol	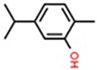	C_10_H_14_O	150.22	Strong fumigant toxicity against the adults of *M. domestica* [90].
	Piperitenone	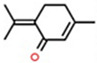	C_10_H_14_O	150.22	Larvicidal and pupicidal activity against *C. quinquefasciatus* [91].
	Thymol	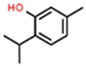	C_10_H_14_O	150.22	Antifeedant on the adult insects of *S. littoralis*, *M. persicae,* and *L. decemlineata*, and toxicity against second-stage juveniles of the phytopathogenic nematode *M. javanica* [73].
	Pulegone	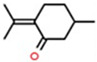	C_10_H_16_O	152.23	Strong fumigant toxicity against the adults of *M. domestica* [90].
	Geranial	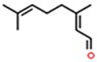	C_10_H_16_O	152.23	Larvicidal and pupicidal activity against *C. quinquefasciatus* [91].
	Borneol	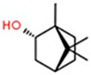	C_10_H_18_O	154.25	Acute toxicity and synergistic effect on the *C. quinquefasciatus* larvae [86].
	Geraniol	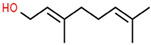	C_10_H_18_O	154.25	Fumigant and contact toxicity, and neurophysiological impacts against *C. lectularius* [77].
	Linalool	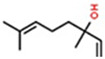	C_10_H_18_O	154.25	The inhibition of acetylcholine esterase and insecticidal activity on *S. oryzae* [87].
	Terpinene-4-ol	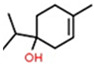	C_10_H_18_O	154.25	The inhibition of acetylcholine esterase and insecticidal activity on *S. oryzae* [87].
	α-Terpineol	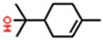	C_10_H_18_O	154.25	Fumigant toxicity on the adults of *S. granarius* [76].
	Piperitenone oxide	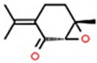	C_10_H_14_O_2_	166.22	Larvicidal activity against *C. pipiens* [92].
	Geranyl acetate	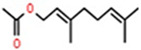	C_12_H_20_O_2_	196.29	Fumigant toxicity on the adults of *S. granarius* [76].
Sesquiterpene hydrocarbon	β-Caryophyllene	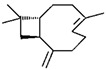	C_15_H_24_	204.35	The inhibition of acetylcholine esterase and insecticidal activity on *S. oryzae* [87].
Sesquiterpenoid	Caryophyllene oxide	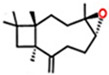	C_15_H_24_O	220.35	Insecticidal effects against the larvae and pupae of fall armyworm (*Spodoptera frugiperda* (Smith)) [93].
Phenylpropanoid	Estragole	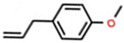	C_10_H_12_O	148.20	Fumigant and contact toxicity, and acetylcholine esterase inhibition activity against *B. germanica* [89].
